# Potentially Functional Genetic Variants in the NRF2 Signaling Pathway Genes are Associated With HBV-related Hepatocellular Carcinoma Survival

**DOI:** 10.7150/jca.88561

**Published:** 2023-10-16

**Authors:** Rongbin Gong, Moqin Qiu, Ji Cao, Zihan Zhou, Yuying Wei, Qiuping Wen, Qiuling Lin, Xiaoxia Wei, Xiumei Liang, Yanji Jiang, Peiqin Chen, Junjie Wei, Shicheng Zhan, Yingchun Liu, Hongping Yu

**Affiliations:** 1 Department of Experimental Research, Guangxi Medical University Cancer Hospital, Nanning 530000, China.; 2 Department of Epidemiology and Health Statistics, School of Public Health, Guangxi Medical University, Nanning 530000, China.; 3 Department of Respiratory Oncology, Guangxi Medical University Cancer Hospital, Nanning 530000, China.; 4 Department of Cancer Prevention and Control, Guangxi Medical University Cancer Hospital, Nanning 530000, China.; 5 Department of Clinical Research, Guangxi Medical University Cancer Hospital, Nanning 530000, China.; 6 Department of Disease Process Management, Guangxi Medical University Cancer Hospital, Nanning 530000, China.; 7 Department of Scientific Research Dept, Guangxi Medical University Cancer Hospital, Nanning 530000, China.; 8 Key Cultivated Laboratory of Cancer Molecular Medicine of Guangxi Health Commission, Guangxi Medical University Cancer Hospital, Nanning 530000, China.; 9 Key Laboratory of Early Prevention and Treatment for Regional High Frequency Tumor (Guangxi Medical University), Ministry of Education, Nanning 530000, China.

**Keywords:** NRF2, HBV-related HCC, SNPs, OS, eQTL

## Abstract

The nuclear factor E2-related factor 2 (NRF2) signaling pathway is one of the most important cell defense pathways. However, it is unclear whether genetic variants in NRF2 signaling pathway genes are associated with the survival of hepatitis B virus (HBV)-related hepatocellular carcinoma (HCC). In the present study, we utilized a new hypothesis-driven approach based on biological pathways to investigate the associations between 17919 single nucleotide polymorphisms (SNPs) in 137 NRF2 signaling pathway genes and the overall survival (OS) of 866 patients with HBV-related HCC. As a result, two independent SNPs with potential biological function were identified to be significantly associated with HBV-related HCC OS: [*SLC2A9* rs28643326 T>C: hazard ratio (HR) = 0.74, 95% confidence interval (95% CI) = 0.62-0.89, *P* < 0.001 and *SLC5A10* rs2472711 G>T: HR = 0.81, 95% CI = 0.71-0.93,* P* = 0.003, respectively]. The expression quantitative trait loci (eQTL) analysis further revealed that the rs28643326 C allele was significantly associated with increased levels of *SLC2A9* mRNA expression (*P* < 0.001), and higher mRNA expression levels of *SLC2A9* in adjacent normal liver tissues were associated with better survival. Although the association between the rs2472711 T allele and the mRNA expression of *SLC5A10* was not statistically significant (*P* = 0.200), the fact that rs2472711 is located at the DNase I hypersensitivity site and is a marker for promoter and enhancer histones also suggests that it may have the function of regulating its corresponding gene expression. In conclusion, genetic variants of NRF2 signaling pathway genes may serve as potential prognostic biomarkers for HBV-related HCC and also provide a solid basis for further mechanistic exploration.

## Introduction

Primary liver cancer is one of the most common malignancies worldwide and has become the second leading cause of cancer deaths in China, with approximately 410,000 new cases and 391,000 deaths in 2020 1. Hepatocellular carcinoma (HCC) is the most common primary liver cancer, accounting for approximately 80% of all cases, and hepatitis B virus (HBV) is the major risk factor, with nearly 84% of HCC patients infected in China 2, 3. Although the level of treatment for HCC has been greatly improved in recent years, the 5-year survival rate for patients with middle-advanced HCC is still pessimistic with only 14.1% 4. Currently, the major clinicopathological variables that are utilized to predict the prognosis of HCC include age, sex, alpha-fetoprotein (AFP) levels, cirrhosis, and tumor stage. Notably, due to individual heterogeneity, even the HCC patients with the same clinical characteristics may have different outcomes, suggesting that genetic susceptibility may have an impact on the prognosis of HCC patients 5. Therefore, it is important to explore proper prognostic biomarkers for HCC.

As the most common type of genetic variant, single nucleotide polymorphisms (SNPs) can regulate gene expression by altering transcriptional activity. To date, the genome-wide association analysis (GWAS) possesses the ability to efficiently identify the SNPs associated with the development and progression of tumors, and a growing number of SNPs have been successively confirmed to be associated with the risk or survival of HCC 6, 7. However, a large number of the SNPs with significant regulatory functions have been overlooked because of the failure to meet the stringent statistical *P* values. With the advent of the post-GWAS era, a new hypothesis-driven approach based on biological pathways has emerged for better investigating the impact of biological pathways and is now widely utilized to identify more SNPs of the pathway genes that have potential function on the HCC survival 8.

The nuclear factor E2-related factor 2 (NRF2) signaling pathway is one of the most important cell defense pathways 9, primarily by regulating gene expression to resist oxidative stress, maintain cellular homeostasis, and play an important protective role in tumorigenesis and development 10. It has been shown that over-activation of the NRF2 signaling pathway enhances cellular resistance to the oxidative stress and electromagnetic waves involved in multiple stages of tumor development by regulating cell cycle and cell motility 11.* NRF2*, a master transcription factor with functions in regulating inflammatory and antiviral responses, has also been shown to act as a tumor suppressor that protects cells from endogenous and exogenous stresses and inhibits carcinogenesis 12, 13.* NRF2* has been found to play an important protective role in various liver diseases, including liver inflammation, liver fibrosis, and hepatocarcinogenesis, through the inhibition of reactive oxygen species (ROS) 14. Additionally, it has been suggested that *NRF2* may regulate several important genes to detoxify and combat oxidative stress in the prevention of carcinogenesis 15. In recent years, a number of SNPs in NRF2 signaling pathway genes have been identified to be associated with the risk and prognosis of pancreatic cancer 16, breast cancer 17, and cholangiocarcinoma 18, further supporting the vital role of the NRF2 pathway playing in cancer susceptibility and prognosis. Unfortunately, little is known about the effect of genetic variants in NRF2 signaling pathway genes on the survival of the HBV-related HCC. Considering the importance of the NRF2 pathway in HCC, based on genotyping data from the Guangxi population in China, we aimed to investigate the associations between SNPs with potential functions in NRF2 signaling pathway genes and the survival of the HBV-related HCC.

## Material and Methods

### Study populations

From June 2007 to December 2017, 866 HCC patients who underwent hepatectomy and were seropositive for hepatitis B surface antigen (HBsAg) were recruited at Guangxi Medical University Cancer Hospital and randomized into discovery and validation groups in a 1:1 ratio. The inclusion and exclusion criteria were as follows: 1) All participants must be pathologically diagnosed HCC patients; 2) All patients must have positive serum HBsAg test results; 3) Patients with surface-positive anti-hepatitis C virus were excluded; 4) Patients with cardiovascular, renal or pulmonary diseases were excluded; 5) Patients who had developed pathological distant metastases, and those who had received preoperative radiotherapy or chemotherapy for hepatectomy were excluded; 6) Patients lacking complete clinical and follow-up information were further excluded. Detailed information has been described in a previous study 19. We gathered demographic and clinical information on all individuals, including age, sex, smoking status, drinking status, AFP level, cirrhosis, embolus, and Barcelona Clinic Liver Cancer (BCLC) stage. In addition, 5 ml of peripheral blood samples were collected from all individuals for genotyping with the Illumina GSA genotyping chip (Infnium Global Screening Array-24 v1.0), and Minimac3 was utilized for imputation based on 1000 Genomes Project (Phase 3 v5) reference population information (https://imputationserver.sph.umich.edu/index.htm). We used overall survival (OS) as the primary endpoint and acquired it through a rigorous follow-up that ended in March 2020. Informed consent was obtained from all patients, and the study was approved by the Ethics Committee of Guangxi Medical University Cancer Hospital (Approval Number: LW2023105).

### Genes and SNPs selection

NRF2 signaling pathway genes were identified in the Molecular Signatures Database (http://software.broadinstitute.org/gsea/msig-db/index.jsp) using “NRF2” as a keyword. After excluding four genes on the X chromosome, 137 genes remained as candidate genes **(Table S1)**. All SNPs within candidate genes and their flanking regions of ± 2 kb were extracted according to the following criteria: genotyping rate ≥ 95%, minimum allele frequency (MAF) ≥ 0.05, and Hardy-Weinberg equilibrium (HWE) *P* value ≥ 1×10-6.

### RNA sequencing

After obtaining consent, we collected 100 pairs HCC tissues and adjacent normal tissues from Guangxi Medical University Cancer Hospital for RNA sequencing to detect differential expression of genes in HCC.

### Statistical analyses

By using the GenABEL package in R software 20, the multivariate Cox proportional hazards regression analysis adjusted for age, sex, smoking status, drinking status, AFP level, cirrhosis, embolus, and BCLC stage was used to calculate the hazard ratio (HR) and 95% confidence interval (95% CI) for all the SNPs in an additive model to assess the associations between SNPs in 137 candidate genes of the NRF2 signaling pathway and the HBV-related HCC OS. Next, to reduce the possibility of false positive results, the Bayesian false discovery probability (BFDP) approach was utilized for multiple testing correction. Only SNPs with a BFDP value ≤ 0.8 were chosen for validation. Subsequently, we included the validated SNPs in a multivariate stepwise Cox model with adjustment for clinical variables to identify independent SNPs and employed additive and dominant genetic models to assess the associations between different genotypes of independent SNPs and the HBV-related HCC OS. In addition, the combination of protective genotypes was conducted to estimate the joint effects of identified SNPs, and Kaplan-Meier survival curves with log-rank tests were used to observe the influence of the joint effect on OS. Then, we further utilized stratified analysis to assess whether the combined effect of protective genotypes on OS was influenced by clinical covariates. Finally, Haploview v4.2 was used to generate Manhattan plots 21, and LocusZoom 22 was used to construct regional association plots.

All statistical analyses were performed using the R software (versions 3.1.3 and 4.2.2), and *P* < 0.05 was considered statistically significant.

### Function prediction

In order to predict the potential function of the identified SNPs, we utilized three available online bioinformatics tools: RegulomeDB (https://www.regulomedb.org/regulome-search/) 23, HaploReg v4.2 (https://pubs.broadinstitute.org/mammals/haploreg/haploreg.php) 24, and SNPinfo (https://manticore.niehs.nih.gov/snpinfo/snpfunc.html) 25. The expression quantitative trait locus (eQTL) analysis was performed to assess the correlation between SNPs and the corresponding genes mRNA expression levels by using the RNA sequencing data of lymphoblastoid cell lines (n = 76) generated from Chinese descendants in the 1000 Genomes Project Han Chinese in Beijing and the data of whole blood (n = 369) and normal liver tissues (n = 220) from the Genotype-Tissue Expression Project (GTEx) database (http://www.gtexportal.org/home/) 26. We further compared the variation of gene mRNA expression levels between HCC tissues and adjacent normal tissues by using the Cancer Genome Atlas (TCGA) database 27 and in-house RNA sequencing data. In addition, the Kaplan-Meier Plotter (https://kmplot.com/analysis/), an online survival analysis software, was used to illustrate the correlation between the corresponding gene mRNA expression levels and the survival in patients with liver cancer. At last, the public database of the cBioPortal for Cancer Genomics was utilized to evaluate the somatic mutational status of genes in HCC.

## Results

### Associations between SNPs in NRF2 pathway genes and HBV-related HCC OS

**Figure 1:**
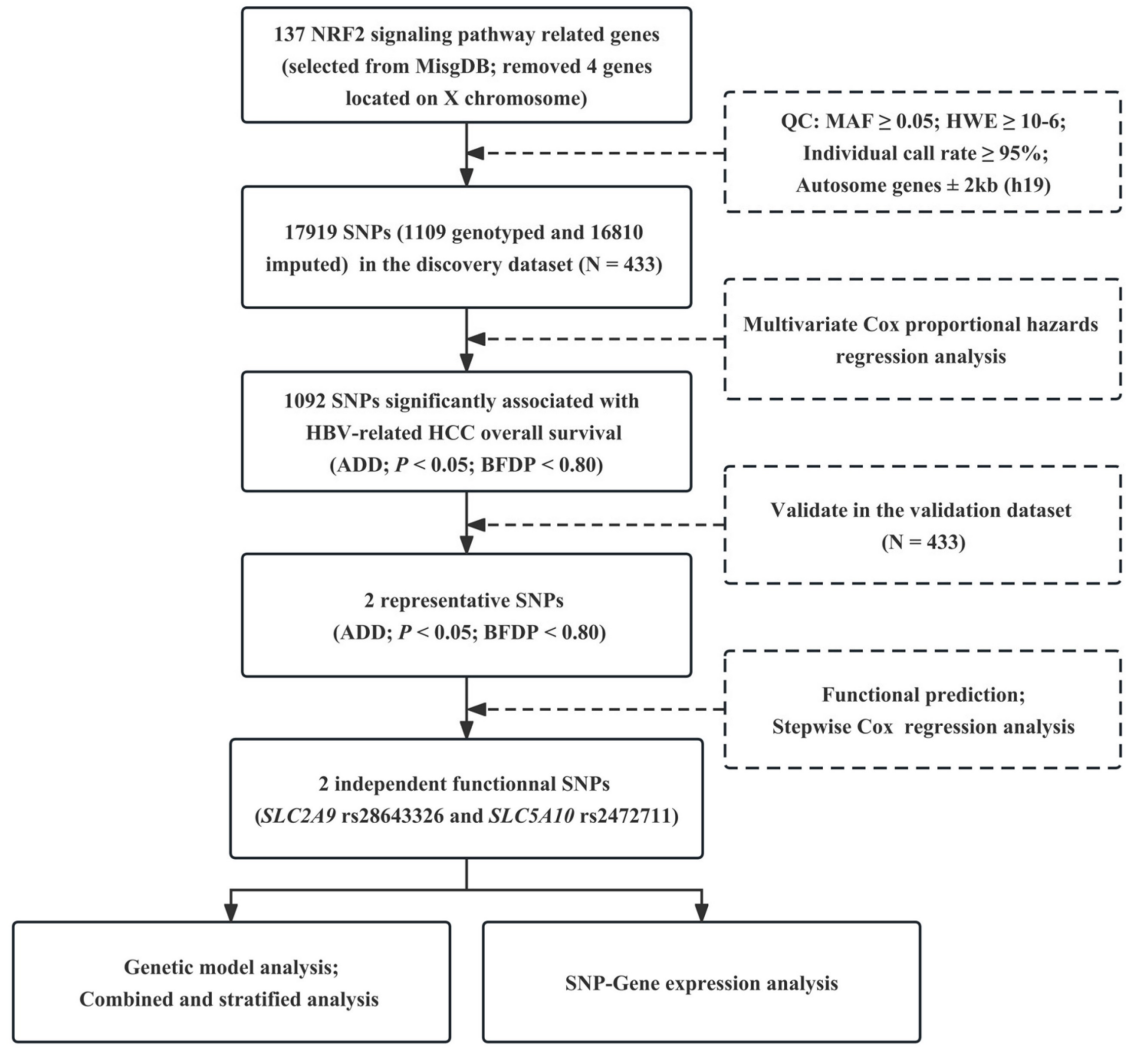
Workflow of the study process. NRF2: nuclear factor E2-related factor 2; MsigDB: Molecular Signatures Database; QC: quality control; MAF: minor allele frequency; HWE: Hardy-Weinberg equilibrium; SNP: single nucleotide polymorphism; HBV: hepatitis B virus; HCC: hepatocellular carcinoma; ADD: additive model; BFDP: Bayesian false-discovery probability.

The workflow of this study is presented in **Figure 1**. As described in the previous study, the demographic and clinical characteristics of 866 patients with the HBV-related HCC were shown **in Table S2**. We extracted 17919 SNPs (1109 genotyped and 16810 imputed) in the 137 NRF2 signaling pathway genes with 2 kb flanking regions from the discovery dataset. In the single locus analysis of the discovery dataset, 1207 SNPs were significantly associated with the OS of the HBV-related HCC in the additive genetic model (*P* < 0.05, BFDP < 0.80), of which rs28643326 in solute carrier family 2, member 9 (*SLC2A9*), and rs2472711 in solute carrier family 5, member 10 (*SLC5A10*) remained statistically significant after further replication in the validation dataset **(Table 1)**. These results were also depicted by Manhattan plots in **Figure S1**.

### Independent SNPs in NRF2 pathway genes were associated with HBV-related HCC OS

The multivariable stepwise Cox regression model was performed with adjustment for clinical variables in the combined dataset to observe whether the identified SNPs were independent predictors for the HCC OS. As a result, two SNPs remained statistically significant **(Table 2)**. Consequently, the results of genetic model analysis in the combined dataset showed that rs28643326 TC/CC and rs2472711 GT/TT were presented to be associated with better prognosis in the HBV-related HCC patients (HR = 0.71, 95% CI = 0.57-0.87, *P* < 0.001 and HR = 0.74, 95% CI = 0.60-0.90, *P* = 0.003, respectively) **(Table 3)**. For illustrative purposes, two SNPs in their corresponding genes with 50kb flanking region are shown in regional association plots (**Figure S2**).

### Combined and stratified analysis of the two independent SNPs

**Figure 2:**
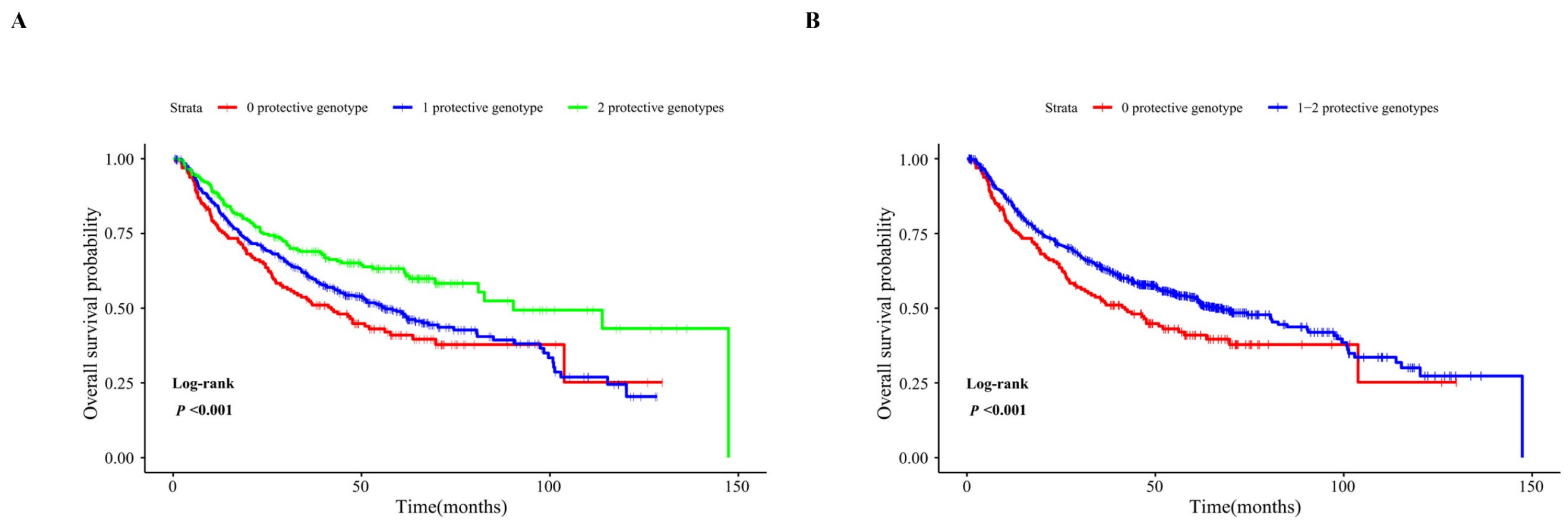
Kaplan-Meier analysis for patients with HBV-related HCC by the combined protective genotypes. Kaplan-Meier analysis for patients with HBV-related HCC by 0, 1, and 2 protective genotypes (A), by 0 and 1-2 protective genotypes (B).

To assess the joint effect of the two independent SNPs on the OS of HBV-related HCC, we combined protective genotypes (*SLC2A9* rs28643326 TC/CC and *SLC5A10* rs2472711 GT/TT) into a genetic score based on the number of protective genotypes (NPGs). All HCC patients were divided into three groups with genetic scores of 0, 1, and 2, respectively. The results indicated that patients with a genetic score of 1 or 2 had a more favorable OS than that with a score of 0 (*P* = 0.026 and < 0.001, respectively), and a higher genetic score was associated with a progressively better survival in the combined dataset (*P*_trend_ < 0.001). Next, all HCC patients were further dichotomized into a low-protection group (score 0) and a high-protection group (score 1-2). We found that the patients in the high-protection group had better survival compared to those in the low-protection group in the combined dataset (HR = 0.67, 95% CI = 0.53-0.85, *P* < 0.001) **(Table 4)**, and Kaplan-Meier survival curves were employed to describe these results **(Figure 2)**. The stratified analysis results showed that the HCC patients in the high-protection group had a favorable prognosis in all the subgroups (*P* < 0.05) except age > 47, female, drinking, AFP ≤ 400ng/ml, and patients with cancer embolus. However, no significant interaction between NPG and clinical variables was found **(Table 5)**.

### In silico functional validation

**Figure 3:**
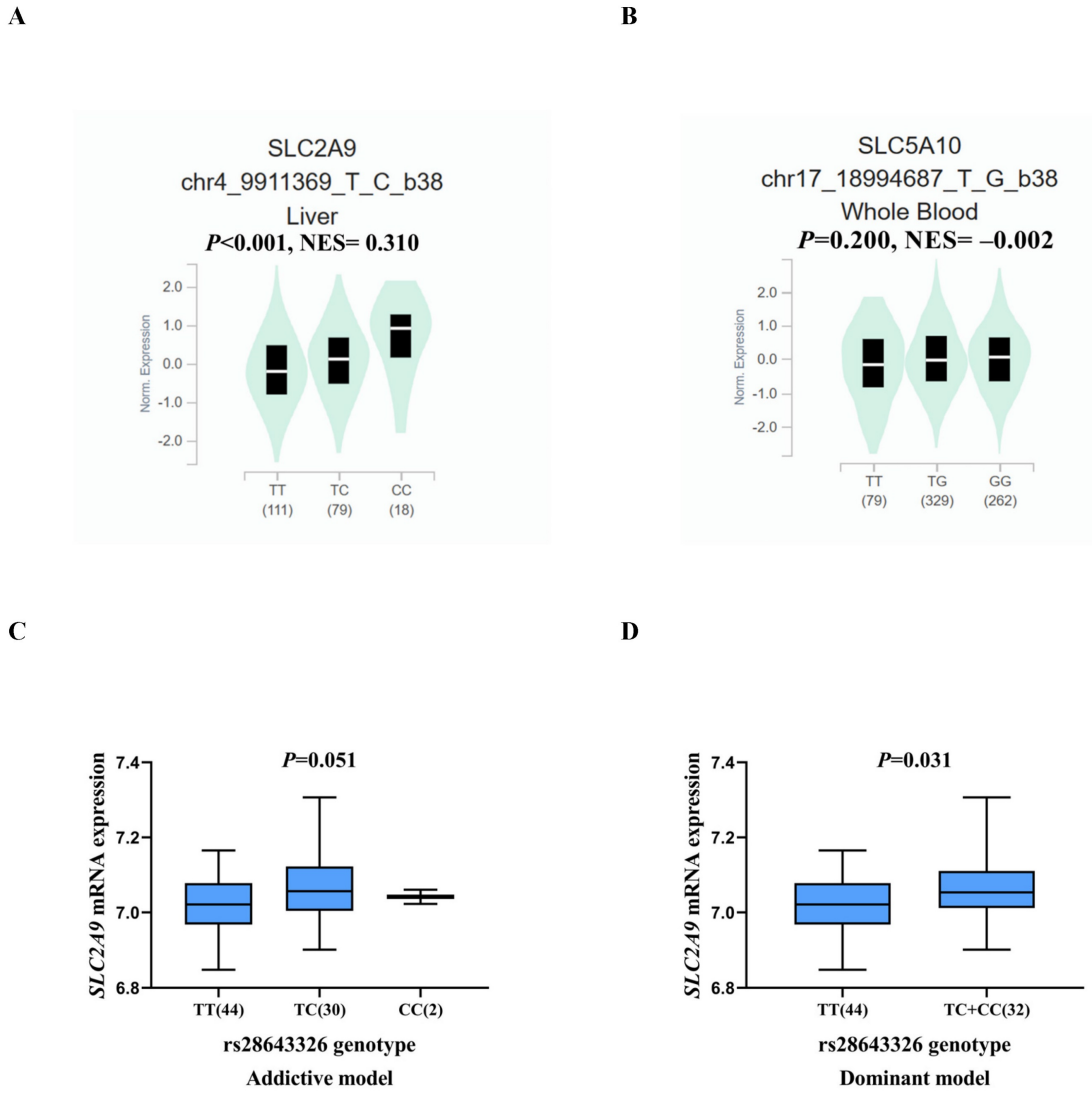
Correlation of the SNPs with mRNA expression. eQTL analysis of* SLC2A9* rs28643326 in liver tissue from the GTEx database (n = 208, *P* < 0.001) (A), eQTL analysis of *SLC5A10* rs2472711 in whole blood from the GTEx database (n = 670, *P* = 0.200) (B), correlation of rs28643326 with *SLC2A9* mRNA expression in lymphoblastoid cells from the 1000 Genomes Project Han Chinese in Beijing in the additive (n = 76, *P* = 0.051) (C) and dominant (n = 76, *P* = 0.031) (D) models. SNPs: single nucleotide polymorphisms; eQTL: expression quantitative trait loci; GTEx: Genotype-Tissue Expression project; NES: normalized effect size; Norm: normalized.

**Figure 4:**
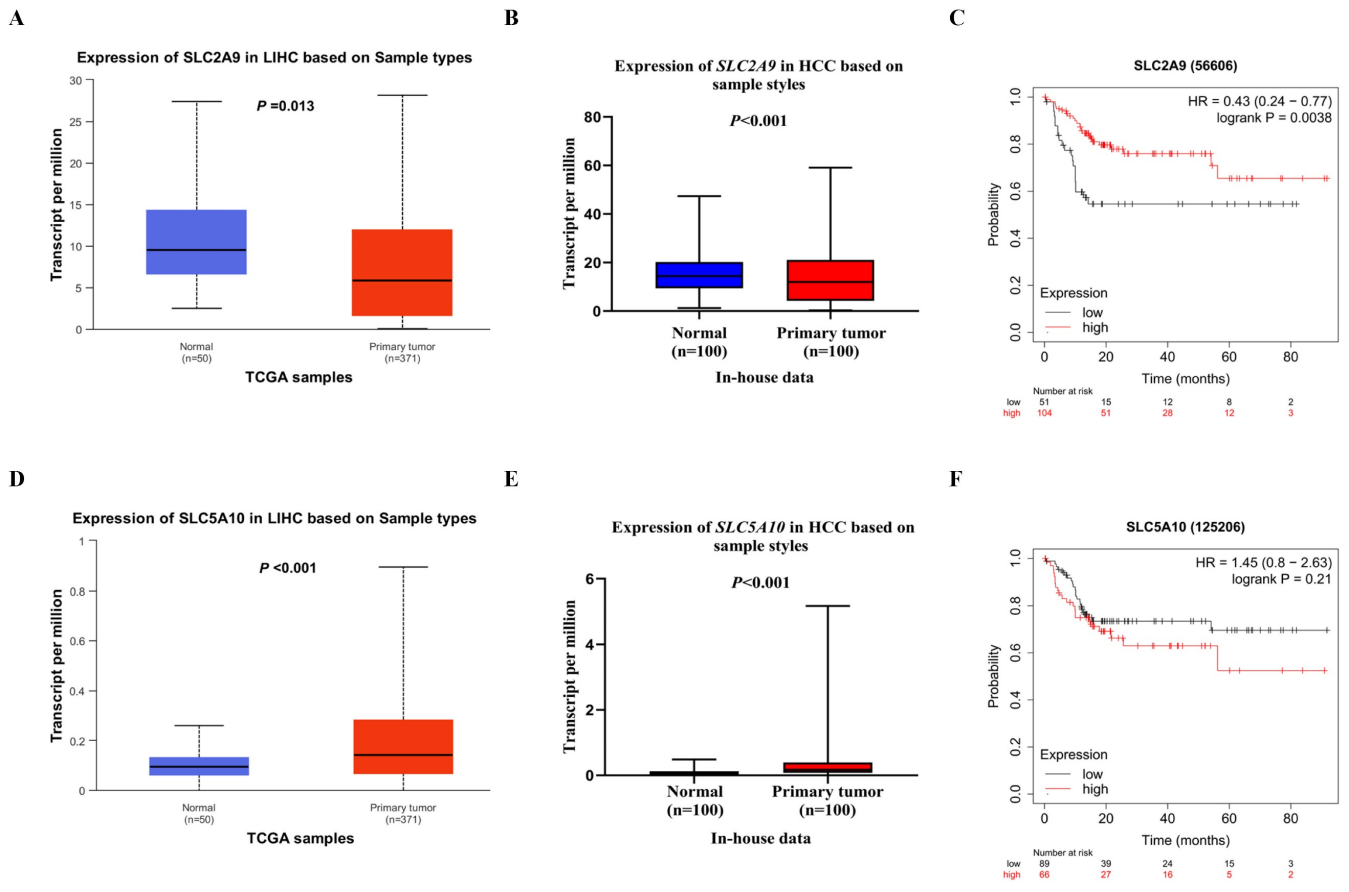
Differential mRNA expression analysis and survival of HCC. The relative expression levels of the *SLC2A9* mRNA in the TCGA database (A) and in-house RNA-Seq data (B), the relative expression levels of the *SLC5A10* mRNA in the TCGA database (D) and in-house RNA-seq data (E), liver cancer patients with high mRNA expression levels of *SLC2A9* had a better OS (C), liver cancer patients with high mRNA expression levels of *SLC5A10* had a worse OS (F). TCGA: the Cancer Genome Atlas; HCC: hepatocellular carcinoma; HR: hazard ratio; LIHC: liver hepatocellular carcinoma; RNA-seq: RNA sequencing; OS: overall survival.

As shown in **Table S3**, biological function annotations showed that *SLC2A9* rs28643326 and *SLC5A10* rs2472711 were both located in the intron region with RegulomeDB scores of 5 and 3a, respectively, which might change multiple motifs. Furthermore, *SLC2A9* rs28643326 was located at a transcription factor binding site (TFBS), and *SLC5A10* rs2472711 was located at 7 DNase I hypersensitive sites and may be markers of promoter or enhancer histones. The eQTL analysis was performed to further explore the potential functions of the two identified SNPs. We found that the rs28643326 C allele was significantly associated with elevated mRNA expression levels of *SLC2A9* in normal liver tissues from the GTEx project (*P* < 0.001, **Figure 3A**), as well as in lymphoblastoid cells from the 1000 Genomes Project Han Chinese in Beijing (*P* = 0.093 for the additive genetic model, **Figure 3C**, and *P* = 0.031 for dominant genetic model,** Figure 3D,** respectively). However, genotyping data for *SLC5A10* rs2472711 in normal liver tissues were not available, and there was no significant correlation between the rs2472711 T allele and mRNA expression levels of *SLC5A10* in whole blood (*P* = 0.200,** Figure 3B**) from the GTEx project. Additionally, gene expression data from the TCGA and in-house RNA-Seq dataset illustrated that the mRNA expression of *SLC2A9* was significantly lower in HCC tissues compared to adjacent normal tissues **(Figure 4A and Figure 4B)**. In contrast, in comparison with adjacent normal tissues, HCC tissues had a higher mRNA expression level of *SLC5A10* **(Figure 4D and Figure 4E)**. Finally, K-M survival curves obtained based on the Kaplan-Meier Plotter website showed that higher expression of *SLC2A9* was significantly associated with improved liver cancer survival (*P* = 0.004, **Figure 4C**), whereas higher expression levels of *SLC5A10* were was associated with a worse survival of liver cancer (*P* = 0.210, **Figure 4F**).

### Mutation analysis

The public database of the cBioPortal for Cancer Genomics was utilized to explore the mutation status of *SLC2A9* and* SLC5A10* in HCC. As shown in **Figure S3**, both genes had very low somatic mutation rates in different HCC datasets (*SLC2A9:* 0.82%, 2/243 in the INSERM; 0.27%, 1/366 in the TCGA PanCancer Atlas study; 0.27%, 1/373 in the TCGA Firehose Legacy study; and 0.43%, 1/231 in the AMC; *SLC5A10*: 0.41%, 1/243 in the INSERM; 0.55%, 2/366 in the TCGA PanCancer Atlas study; 0.54%, 2/373 in the TCGA Firehose Legacy study; and 0.43%, 1/231 in the AMC). Thus, it was evident that the mutations in *SLC5A10* and *SLC2A9* had negligible effects on their expression levels, supporting functional genetic variants as the key influencing factor leading to the imbalance of *SLC2A9* and *SLC5A10* expression in HCC.

## Discussion

In the present study, we evaluated the associations between 17919 SNPs in 137 NRF2 signaling pathway genes and HBV-related HCC OS. Two independent and potentially functional SNPs (*SLC2A9* rs28643326 T>C and *SLC5A10* rs2472711 G>T) have been demonstrated to reduce the risk of death in the patients with HBV-related HCC after hepatectomy. Importantly, an increased number of protective genotypes of these two independent SNPs were significantly associated with better HCC OS. Further eQTL analysis results indicated that the rs28643326 C allele predicted an increased mRNA expression of *SLC2A9*. Moreover, we found that higher mRNA expression levels of *SLC2A9* in adjacent normal liver tissues were associated with better survival, whereas higher mRNA expression levels of* SLC5A10* in HCC tissues were associated with poorer survival. These evidences suggested that these two SNPs in the NRF2 signaling pathway genes may serve as the prognostic biomarkers for HBV-related HCC, especially the *SLC2A9* rs28643326.

*SLC2A9* (also known as GLUT9), located on chromosome 4p16.1, is a member of the glucose transporter family, which plays a key role in cellular metabolism mainly by transporting glucose and fructose 28, 29. A study revealed that *SLC2A9* mRNA expression levels were significantly lowered in a variety of cancer tissues, including kidney, prostate, and testicular cancers. Meantime, lower *SLC2A9* mRNA levels were significantly associated with poorer survival in the patients with gastric cancer 30. In addition, it was reported that *SLC2A9* might be a novel tumor suppressor gene that induced apoptosis in HCC cells by inhibiting the expression of caspase 3, and high expression of *SLC2A9* could effectively inhibit the proliferation of HCC cells 31, suggesting that *SLC2A9* may have an important biological role in HCC.

Consistent with previous reports, in this study, we have observed that the rs28643326 C allele was associated with an increased mRNA expression level of *SLC2A9*, and the *SLC2A9* mRNA expression levels were significantly higher in adjacent normal tissues than in HCC tissues, and their high expression levels were associated with a more favorable HCC OS. Remarkably, rs28643326 is located at the transcription factor binding site that may affect *SLC2A9* expression by enhancing transcriptional activity, which further supports the biological plausibility of the findings. Thus, we further validated that *SLC2A9* played a carcinogenic role in HCC biology and that *SLC2A9* rs28643326 might be an important factor affecting the prognosis of HCC.

*SLC5A10* is located on chromosome 17p11.2 and encodes a protein that may function as a sodium-dependent mannose and fructose co-transporter. Previous research reported that *SLC5A10* rs2257609 C>T affected the prognosis of early-stage non-small-cell lung cancer by influencing the expression of *DRG2* 32. Unfortunately, few studies have revealed the role of *SLC5A10* in HCC. In the present study, we have found that the rs2472711 T allele is capable of reducing the risk of death in patients with HBV-related HCC, and rs2472711 is located at DNase I hypersensitive sites, which also suggests that it may have the function of regulating its corresponding gene expression. However, due to the lack of data supporting the correlation between the rs2472711 T allele and *SLC5A10* mRNA expression levels, more investigations are needed to prove whether the molecular mechanisms of the observed associations are due to biological processes other than affecting the expression of the corresponding gene at the transcriptional level.

Noticeably, some limitations of this study cannot be ignored. Firstly, all subjects in this study were recruited from Southern China, and thus our results may not be generalizable to other ethnic populations due to differences in genetic variation across ethnic groups. Secondly, the sample size of this study is relatively small, and all patients were enrolled at Guangxi Medical University Cancer Hospital, and thus more studies with a larger sample size from multi-centers are urgently needed to validate our results. Finally, further biological and functional experiments for these two independent SNPs should be performed to fully understand their exact molecular mechanisms in HCC.

## Conclusions

In conclusion, we found that two independent functional SNPs (*SLC2A9* rs28643326 T>C and *SLC5A10* rs2472711 G>T) were significantly associated with HBV-associated HCC OS, suggesting that these two SNPs may serve as potential biomarkers for predicting HCC prognosis. It is likely that the *SLC2A9* rs28643326 C allele elevates the expression of the corresponding gene by affecting transcriptional activity. Our results also provide a solid foundation for further functional studies to identify the molecular mechanisms underlying the observed significant association between genetic variants of NRF2 signaling pathway genes and HCC prognosis.

## Supplementary Material

Supplementary figures and tables.

## Figures and Tables

**Table 1:** Associations of two validated significant SNPs with HBV-related HCC OS in the discovery, validation, and combined dataset.

SNP	Chr	Gene	Allele^a^	Discovery dataset (N = 433)				Validation dataset (N = 433)				Combined dataset (N = 866)			
				EAF	HR (95% CI)	*P* ^b^	BFDP	EAF	HR (95% CI)	*P* ^b^	BFDP	EAF	HR (95% CI)	*P* ^b^	BFDP
rs28643326	4	*SLC2A9*	T>C	0.22	0.73 (0.57-0.94)	0.014	0.66	0.20	0.75 (0.57-0.97)	0.029	0.76	0.21	0.74 (0.62-0.89)	0.001	0.23
rs2472711	17	*SLC5A10*	G>T	0.45	0.80 (0.66-0.97)	0.022	0.75	0.49	0.81 (0.67-0.98)	0.032	0.79	0.47	0.81 (0.71-0.93)	0.003	0.38

**Abbreviations:** SNPs: single nucleotide polymorphisms; HBV: hepatitis B virus; HCC: hepatocellular carcinoma; OS: overall survival; EAF: effect allele frequency; HR: hazards ratio; 95% CI: 95% confidence interval; BFDP: Bayesian false-discovery probability. ^a^ Referring allele/effect allele. ^b^ Multivariate Cox proportional hazards regression analysis was adjusted for age, sex, smoking status, drinking status, AFP level, cirrhosis, embolus, and BCLC stage.

**Table 2:** Two independent predictors of OS obtained from stepwise Cox regression analysis in the combine dataset

Characteristics	Category	Frequency	HR (95% CI)	*P* ^a^
Age (year)	≤47	434	1.00	
	>47	432	0.80 (0.66-0.97)	0.026
Sex	Female	106	1.00	
	Male	760	1.28 (0.93-1.77)	0.135
AFP level (ng/ml)	≤400	522	1.00	
	>400	344	1.30 (1.06-1.60)	0.011
Embolus	No	636	1.00	
	Yes	230	1.72 (1.36-2.18)	<0.001
BCLC stage	0/A	427	1.00	
	B/C	439	2.02 (1.59-2.57)	<0.001
*SLC2A9* rs28643326 T>C	TT/TC/CC	534/295/37	0.73 (0.61-0.88)	<0.001
*SLC5A10* rs2472711 G>T	GG/GT/TT	261/397/208	0.81 (0.71-0.92)	0.002

**Abbreviations:** OS: overall survival; HR: hazards ratio; 95% CI: 95% confidence interval; AFP: alpha-fetoprotein; BCLC: Barcelona Clinic Liver Cancer. ^a^ Stepwise Cox regression analysis was adjusted for age, sex, smoking status, drinking status, AFP level, cirrhosis, embolus, and BCLC stage.

**Table 3:** Associations between two independent SNPs and OS of HBV-related HCC in different genetic models.

Genotype	Discovery dataset (N = 433)				Validation dataset (N = 433)				Combined dataset (N = 866)			
	All	Death (%)	HR (95% CI)	*P* ^a^	All	Death (%)	HR (95% CI)	*P* ^a^	All	Death (%)	HR (95% CI)	*P* ^a^
*SLC2A9* rs28643326 T>C												
TT	263	134 (51.0)	1.00		271	148 (54.6)	1.00		534	282 (52.8)	1.00	
TC	147	60 (40.8)	0.79 (0.58-1.08)	0.140	148	64 (43.2)	0.66 (0.48-0.89)	0.007	295	124 (42.0)	0.72 (0.58-0.89)	0.002
CC	23	6 (26.1)	0.41 (0.18-0.93)	0.032	14	7 (50.0)	1.00 (0.47-2.15)	0.992	37	13 (35.1)	0.63 (0.36-1.10)	0.101
Trend test				0.014				0.029				0.001
TT	263	134 (51.0)	1.00		271	148 (54.6)	1.00		534	282 (52.8)	1.00	
TC+CC	170	66 (38.8)	0.73 (0.54-0.98)	0.036	162	71 (43,8)	0.68 (0.51-0.91)	0.010	332	137 (41.3)	0.71 (0.57-0.87)	<0.001
*SLC5A10* rs2472711 G>T												
GG	141	75 (53.2)	1.00		120	65 (54.2)	1.00		261	140 (53.6)	1.00	
GT	191	85 (44.5)	0.75 (0.54-1.03)	0.071	206	105 (51.0)	0.76 (0.56-1.04)	0.092	397	190 (47.9)	0.77 (0.62-0.96)	0.022
TT	101	40 (39.6)	0.65 (0.44-0.96)	0.030	107	49 (45.8)	0.67 (0.46-0.97)	0.035	208	89 (42.8)	0.67 (0.51-0.88)	0.003
Trend test				0.022				0.032				0.003
GG	141	75 (53.2)	1.00		120	65 (54.2)	1.00		261	140 (53.6)	1.00	
GT+TT	292	125 (42.8)	0.71 (0.53-0.95)	0.022	313	154 (49.2)	0.73 (0.54-0.98)	0.035	605	279 (46.1)	0.74 (0.60-0.90)	0.003

**Abbreviations:** SNPs: single nucleotide polymorphisms; OS: overall survival; HBV: hepatitis B virus; HCC: hepatocellular carcinoma; HR: hazards ratio; 95% CI: 95% confidence interval. ^a^ Multivariate Cox proportional hazards regression analysis was adjusted for age, sex, smoking status, drinking status, AFP level, cirrhosis, embolus, and BCLC stage.

**Table 4:** Combined protective genotypes of the two independent SNPs and OS of HBV-related HCC in the combined dataset

NPGs^a^	Combined dataset (N = 866)		Multivariable analysis	
	All	Death (%)	HR (95% CI)	*P* ^b^
0	161	91 (56.5)	1.00	
1	473	240 (50.7)	0.76 (0.59-0.97)	0.026
2	232	88 (37.9)	0.51 (0.38-0.69)	<0.001
Trend test				<0.001
0	161	91 (56.5)	1.00	
1-2	705	328 (46.5)	0.67 (0.53-0.85)	<0.001

**Abbreviations:** SNPs: single nucleotide polymorphisms; OS: overall survival; HBV: hepatitis B virus; HCC: hepatocellular carcinoma; HR: hazards ratio; 95% CI: 95% confidence interval; NPGs: number of protective genotypes. ^a^ Protective genotypes were* SLC2A9* rs28643326 TC/CC and *SLC5A10* rs2472711 GT/TT. ^b^ Multivariate Cox proportional hazards regression analysis was adjusted for age, sex, smoking status, drinking status, AFP level, cirrhosis, embolus, and BCLC stage.

**Table 5:** Stratified analysis of combined protective genotypes with OS of HBV-related HCC

Characteristics	NPG 0		NPGs 1-2		Multivariable analysis		
	All	Death (%)	All	Death (%)	HR (95% CI)	*P* ^a^	*P*_inter_^b^
Age (year)							0.061
≤47	81	55 (67.9)	353	178 (50.4)	0.57 (0.42-0.77)	<0.001	
>47	80	36 (45.0)	352	150 (42.6)	0.85 (0.58-1.22)	0.373	
Sex							0.299
Female	20	12 (60.0)	86	30 (34.9)	0.53 (0.26-1.09)	0.083	
Male	141	79 (56.0)	619	298 (48.1)	0.67 (0.52-0.87)	0.002	
Smoking status							0.724
No	109	64 (58.7)	436	204 (46.8)	0.72 (0.54-0.96)	0.025	
Yes	52	27 (51.9)	269	124 (46.1)	0.57 (0.37-0.88)	0.011	
Drinking status							0.499
No	125	71 (56.8)	489	221 (45.2)	0.66 (0.50-0.86)	0.003	
Yes	36	20 (55.6)	216	107 (49.5)	0.68 (0.41-1.11)	0.122	
AFP level (ng/ml)							0.060
≤400	94	45 (47.9)	428	187 (43.7)	0.76 (0.55-1.06)	0.100	
>400	67	46 (68.7)	277	141 (50.9)	0.58 (0.41-0.81)	0.002	
Cirrhosis							0.424
No	72	38 (52.8)	318	146 (45.9)	0.66 (0.45-0.95)	0.026	
Yes	89	53 (59.6)	387	182 (47.0)	0.70 (0.51-0.95)	0.023	
Embolus							0.831
No	124	62 (50.0)	512	198 (38.7)	0.67 (0.50-0.90)	0.008	
Yes	37	29 (78.4)	193	130 (67.4)	0.68 (0.44-1.04)	0.076	
BCLC stage							0.658
0/A	82	33 (40.2)	345	113 (32.8)	0.66 (0.45-0.98)	0.042	
B/C	79	58 (73.4)	360	215 (59.7)	0.67 (0.50-0.90)	0.008	

**Abbreviations:** OS: overall survival; HBV: hepatitis B virus; HCC: hepatocellular carcinoma; NPGs: number of protective genotypes; HR: hazards ratio; 95% CI: 95% confidence interval; *P*_inter:_ *P*-value for interaction. ^a^ Multivariate Cox proportional hazards regression analysis was adjusted for age, sex, smoking status, drinking status, AFP level, cirrhosis, embolus, and BCLC stage. ^b^ *P*-value for multiplicative interaction analysis between variables and NPG.

## Abbreviations

NRF2: nuclear factor E2-related factor 2
HBV: hepatitis B virus
HCC: hepatocellular carcinoma
SNPs: single nucleotide polymorphisms
OS: overall survival
eQTL: expression quantitative trait loci
AFP: alpha-fetoprotein
GWAS: genome-wide association analysis
ROS: reactive oxygen species
HBsAg: hepatitis B surface antigen
BCLC: Barcelona Clinic Liver Cancer
MAF: minimum allele frequency
HWE: Hardy-Weinberg equilibrium
HR: hazard ratio
95% CI: 95% confidence interval
BFDP: Bayesian false discovery probability
GTEx: Genotype-Tissue Expression Project
TCGA: the Cancer Genome Atlas
NPGs: number of protective genotypes
TFBS: transcription factor binding site
