# Supplementary material for: Potentially Functional Genetic Variants in the NRF2 Signaling Pathway Genes are Associated With HBV-related Hepatocellular Carcinoma Survival
Source: J Cancer. 2023 Oct 16;14(18):3387–96. doi: 10.7150/jca.88561 (PMC10647191; doi:10.7150/jca.88561)
Supplement: Supplementary file 1 — Supplementary figures and tables. [file jcav14p3387s1.pdf]

List of Supplementary Information:

Figure S1. Manhattan plot.

Figure S2. Regional association plots of two independent SNPs.

Figure S3. Mutation frequency of *SLC2A9* and *SLC5A10* in hepatocellular carcinoma.

Table S1. List of 137 selected genes in the NRF2 signaling pathway from the Molecular Signatures Database.

Table S2. Associations of demographics and clinical characteristics with HBV-related HCC OS in the discovery, validation, and combined dataset.

Table S3. Functional annotation of two significant SNP.

A

B

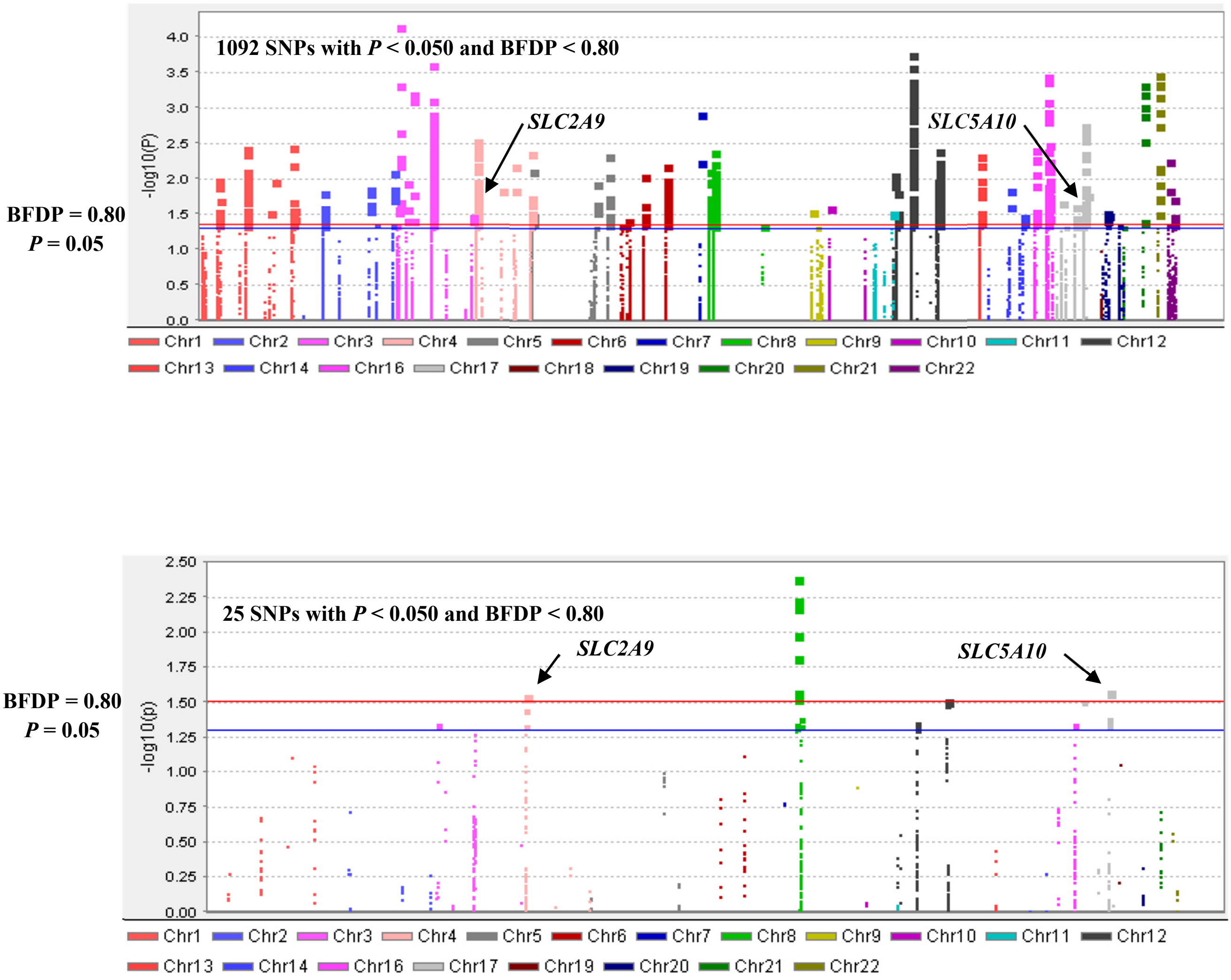

Figure S1. Manhattan plot.

Manhattan plot of 17919 SNPs in the NRF2 signaling pathway genes in the discovery dataset (A), Manhattan plot of 1092 SNPs in the validation dataset (B), the horizontal blue line indicates P = 0.05 and the red line indicates BFDP = 0.80. SNPs: single nucleotide polymorphisms; BFDP: Bayesian false-discovery probability.

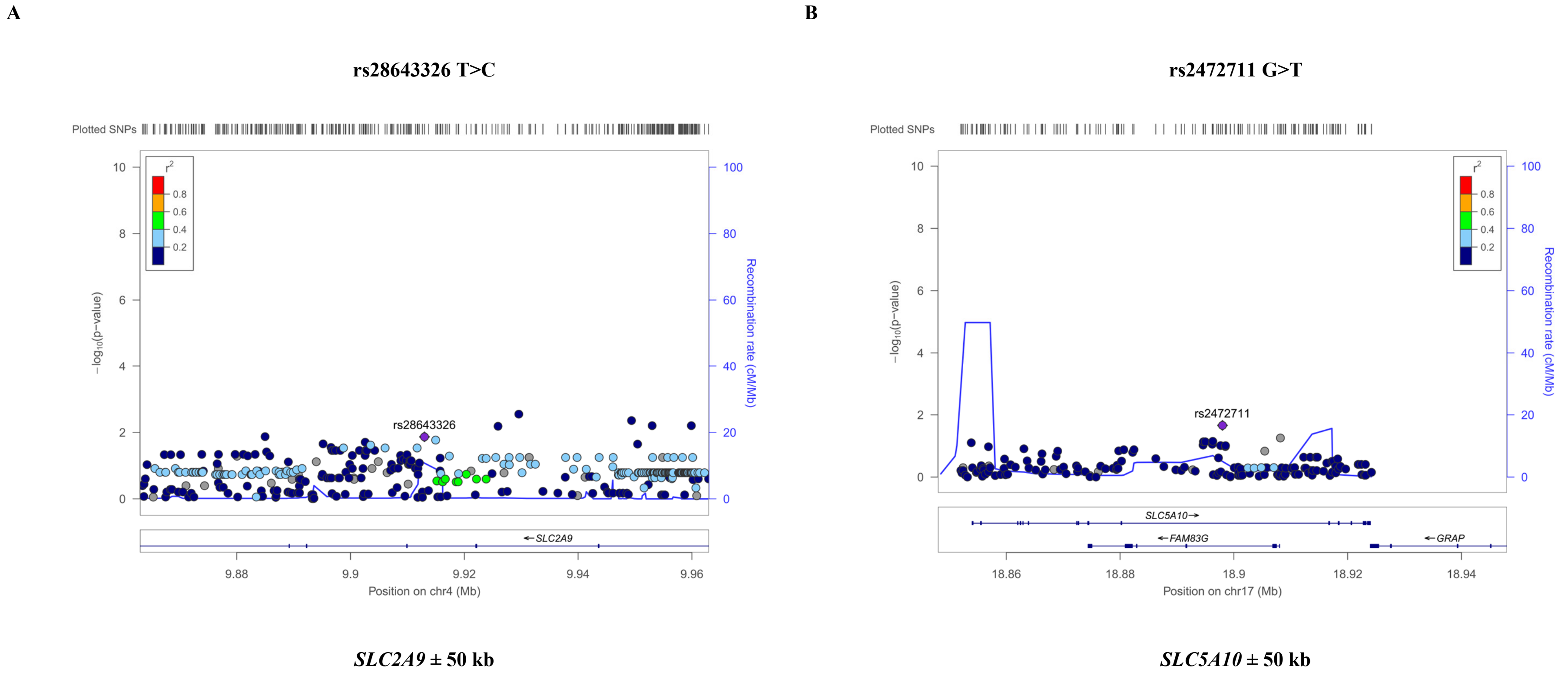

Figure S2. Regional association plots of two independent SNPs**.**

Regional association plot of the *SLC2A9* rs28643326 within regions of ±50 kb (A), regional association plot of the *SLC5A10* rs2472711 within regions of ±50 kb (B).

A

SLC2A9

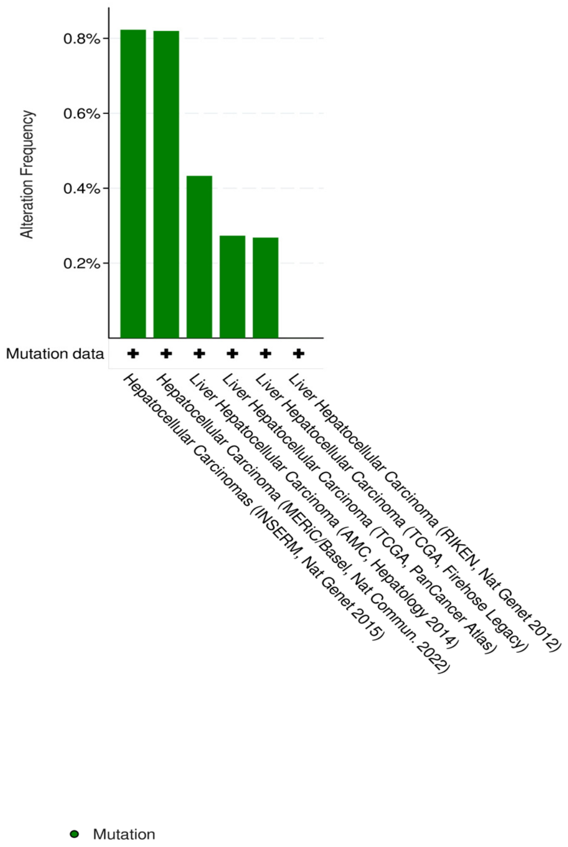

B

SLC5A10

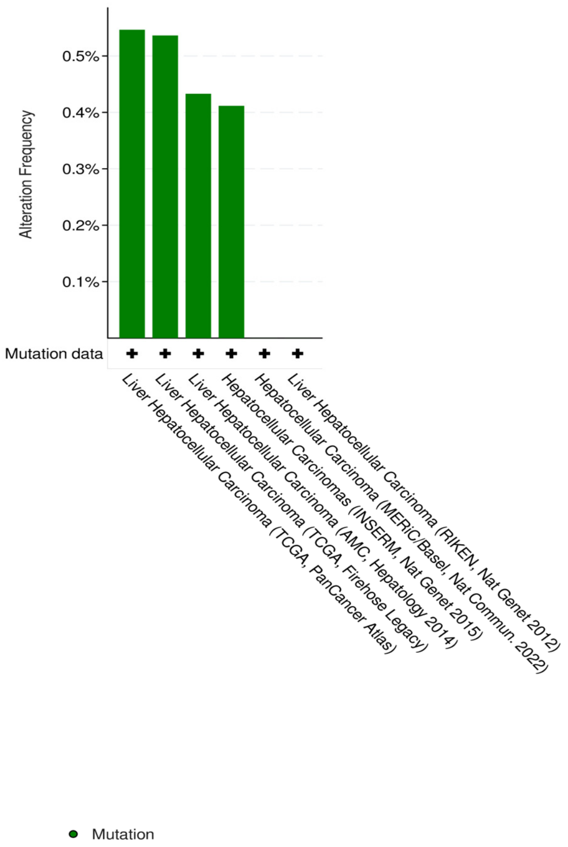

Figure S3. Mutation frequency of *SLC2A9* and *SLC5A10* in hepatocellular carcinoma.

Mutation frequency of *SLC2A9* (A) and *SLC5A10* (B) in hepatocellular carcinoma using the online database of the cBioPortal for Cancer Genomics (http://www.cbioportal.org/).

Table S1. List of 137 selected genes in the NRF2 signaling pathway from the Molecular Signatures Database.

Contributor	Name of geneset	Selected genes
Reactome	WP_NRF2_PATHWAY	*ABCC5, ABCC4, CES1, SLC2A6, SLC6A14, TXNRD3, SLC2A13, SLC5A11, ABCC2, **SLC5A10**, ADH7, SRXN1, SLC2A14, SLC2A12, CYP2A6, SLC2A7, CYP4A11, SLC5A12, SLC5A8, NQO1, AGER, HBEGF, EGR1, EPHA2, SLC5A9, SLC39A11, EPHA3, ALDH3A1, SLC39A12, CES5A, GSTA5, FGF13, PTGR1, CES3, SLC39A14, SLC7A11, MAFF, FTH1, FTL, G6PD, SLC39A6, GGT1, SLC39A1, GCLC, GCLM, SLC39A5, CES4A, GPX2, GPX3, SLC6A16, GSR, GSTA1, GSTA2, GSTA3, GSTA4, GSTM1, GSTM2, GSTM3, GSTM4, GSTM5, GSTP1, GSTT2, SLC39A3, SLC39A2, SLC2A8, HGF, NRG1, HMOX1, HSPA1A, HSP90AA1, HSP90AB1, DNAJB1,SLC6A19, SLC6A18, SLC6A17, MAFG, ME1, MGST2, MGST3, NFE2L2, PRDX1, PDGFB, PGD, SERPINA1, UGT1A7, UGT1A6, UGT1A9, UGT1A4, UGT1A1, PPARD, SLC6A20, SLC6A15, SLC39A9, SLC39A4, **SLC2A9**, SLC39A10, SLC5A7, RXRA, SLC39A8, BLVRB, SLC2A1, SLC2A2, SLC2A3, SLC2A4, SLC2A5, SLC5A1, SLC5A2, SLC5A3, SLC5A4, SLC5A5, SLC6A1, SLC6A2, SLC6A3, SLC6A4, SLC6A6, SLC6A7, SLC6A8, SLC6A9, SLC6A11, SLC6A13, SLC2A11, SOD3, TGFA, TGFB1, TGFB2, TGFBR2, TXN, TXNRD1, UGT2B7, SLC39A7, SLC2A10, ABCC3, CBR1, CBR3, CES2, SQSTM1, SLC5A6, SLC39A13, SLC6A5, PRDX6, KEAP1*
Genes removed		Four genes located on the X chromosome were removed
Total genes		137

Keyword: NRF2

Organism: Homo sapiens

Website: http://www.gsea-msigdb.org/gsea/msigdb/search.jsp

Table S2. Associations of demographics and clinical characteristics with HBV-related HCC OS in the discovery, validation, and combined dataset.

Characteristics	Discovery dataset (N = 433)				Validation dataset (N = 433)				Combined dataset (N = 866)			
	All	Death (%)	HR (95% CI)	P^a	All	Death (%)	HR (95% CI)	P^a	All	Death (%)	HR (95% CI)	P^a
Age (year)												
≤47	220	117 (53.2)	1.00		214	116 (54.2)	1.00		434	233 (53.7)	1.00	
>47	213	83 (39.0)	0.73 (0.55-0.98)	**0.036**	219	103 (47.0)	0.88 (0.67-1.15)	0.349	432	186 (43.1)	0.81 (0.66-0.99)	**0.036**
Sex												
Female	53	22 (41.5)	1.00		53	20 (37.7)	1.00		106	42 (39.6)	1.00	
Male	380	178 (46.8)	1.41 (0.87-2.27)	0.164	380	199 (52.4)	1.22 (0.76-1.97)	0.416	760	377 (49.6)	1.26 (0.90-1.76)	0.176
Smoking status												
No	254	121 (47.6)	1.00		291	147 (50.5)	1.00		545	268 (49.2)	1.00	
Yes	179	79 (44.1)	1.06 (0.73-1.53)	0.769	142	72 (50.7)	0.89 (0.62-1.27)	0.509	321	151 (47.0)	0.91 (0.71-1.17)	0.475
Drinking status												
No	296	137 (46.3)	1.00		318	155 (48.7)	1.00		614	292 (47.6)	1.00	
Yes	137	63 (46.0)	0.81 (0.55-1.19)	0.275	115	64 (55.7)	1.28 (0.89-1.85)	0.183	252	127 (50.4)	1.08 (0.84-1.41)	0.541
AFP level (ng/ml)												
≤400	258	103 (39.9)	1.00		264	129 (48.9)	1.00		522	232 (44.4)	1.00	
>400	175	97 (55.4)	1.56 (1.16-2.11)	**0.004**	169	90 (53.3)	1.08 (0.82-1.43)	0.581	344	187 (54.4)	1.29 (1.05-1.57)	**0.015**
Cirrhosis												
No	208	96 (45.2)	1.00		182	88 (48.4)	1.00		390	184 (47.2)	1.00	
Yes	225	104 (46.2)	0.97 (0.73-1.29)	0.829	251	131 (52.2)	1.07 (0.82-1.41)	0.614	476	235 (49.4)	1.04 (0.85-1.26)	0.702
Embolus												
No	319	122 (38.2)	1.00		317	138 (43.5)	1.00		636	260 (40.1)	1.00	
Yes	114	78 (68.4)	1.87 (1.32-2.65)	**<0.001**	118	81 (68.6)	1.75 (1.26-2.43)	**<0.001**	230	159 (69.1)	1.74 (1.37-2.21)	**<0.001**
BCLC stage												
0/A	225	72 (32.0)	1.00		202	74 (36.6)	1.00		427	146 (34.2)	1.00	
B/C	208	128 (61.5)	2.02 (1.42-2.85)	**<0.001**	231	145 (62.8)	1.90 (1.36-2.65)	**<0.001**	439	273 (62.2)	1.98 (1.56-2.52)	**<0.001**

Abbreviation: OS: overall survival; HBV: hepatitis B virus; HCC: hepatocellular carcinoma; HR: hazards ratio; 95% CI: 95% confidence interval; AFP: alpha-fetoprotein; BCLC: Barcelona Clinic Liver Cancer.

[a] Multivariate Cox proportional hazards regression analysis was adjusted for age, sex, smoking status, drinking status, AFP level, cirrhosis, embolus, and BCLC stage.

Table S3. Functional annotation of two significant SNPs.

SNP	Gene	Location	SNPinfo[a]	Regulome DB score[b]	Haploreg V4.2[c]				
					Promoter histone marks	Enhancer histone marks	DNase	Motifs changed	Selected eQTL hits
rs28643326	*SLC2A9*	intronic	TFBS	5	-	GI	-	GATA, SREBP	1 hit
rs2472711	*SLC5A10*	intronic	-	3a	LNG	9 tissues	7 tissues	11 altered motifs	1 hit

Abbreviation: SNPs: single nucleotide polymorphisms; TFBS: transcription factor binding site; DNase: Deoxyribonuclease; eQTL: expression quantitative trait loci.

a http://snpinfo.niehs.nih.gov/snpinfo/snpfunc.htm.

[b] http://www.regulomedb.org/index.

[c] http://archive.broadinstitute.org/mammals/haploreg/haploreg.php.
